# *Arabidopsis* multiomics reveals the role of autophagy in root microbiome assembly

**DOI:** 10.1002/imo2.28

**Published:** 2024-09-05

**Authors:** Shan Cheng, Yunfeng Shi, Weiming Hu, Fen Liu

**Affiliations:** ^1^ Lushan Botanical Garden Jiangxi Province and Chinese Academy of Sciences Jiujiang China

## Abstract

Upon mutation of the core autophagy protein ATG5, disrupted autophagy pathways result in alterations in several biological processes important for plant‐root microbe interaction mechanisms, including the expression of cell wall‐ and defense‐related proteins and the secretion of root metabolites, all of which affect the root microbial community diversity.

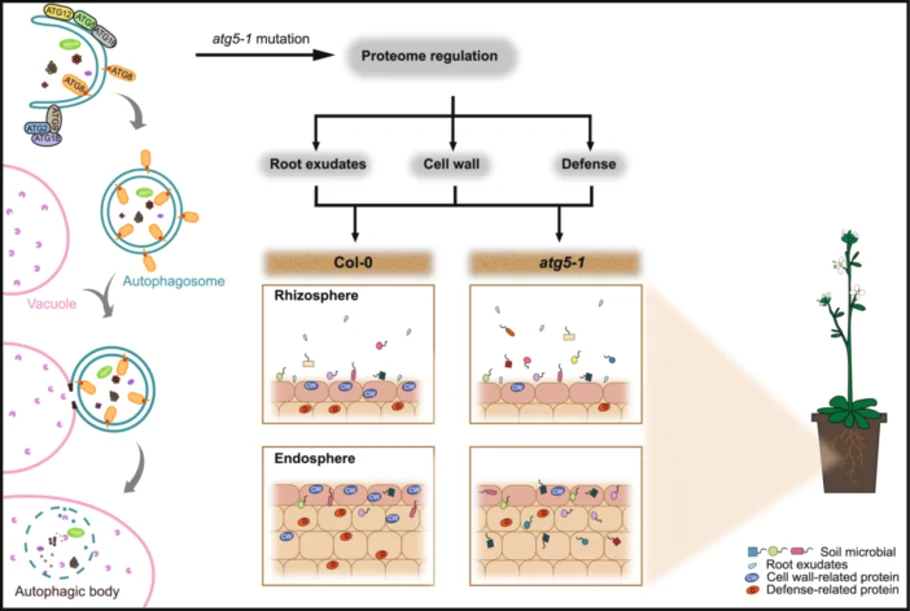

## ETHICS STATEMENT

No animals or humans were involved in this study.

To the editor,

Autophagy, which means “self‐eating,” is a conserved eukaryotic mechanism for the degradation and recycling of cytoplasmic material, including proteins, lipid bodies, nucleic acid aggregates, and even damaged organelles [1, 2]. Under normal conditions, autophagy is mainly used to maintain cellular homeostasis, but increasing autophagy activity allows adaptation to stressful conditions caused by a large variety of environmental cues [3, 4]. In previous research, there has been widespread attention to the physiological role of autophagy in plant growth and development, as well as its regulation of tolerance to various abiotic and biotic stresses [5, 6, 7]. The root microbiota plays an important role in plant nutrition, development, and immunity, and is considered the second genome of plants [8, 9, 10, 11, 12, 13]. However, whether plant autophagy regulates root microorganisms is unknown. In this study, we applied 16S rRNA amplicon and metagenomic approaches to investigate how autophagy affects the assembly and ecological functions of plant‐related microbiomes. Root proteomics and metabolomics were used to evaluate the effects of what on plants in detail.

**Figure 1:**
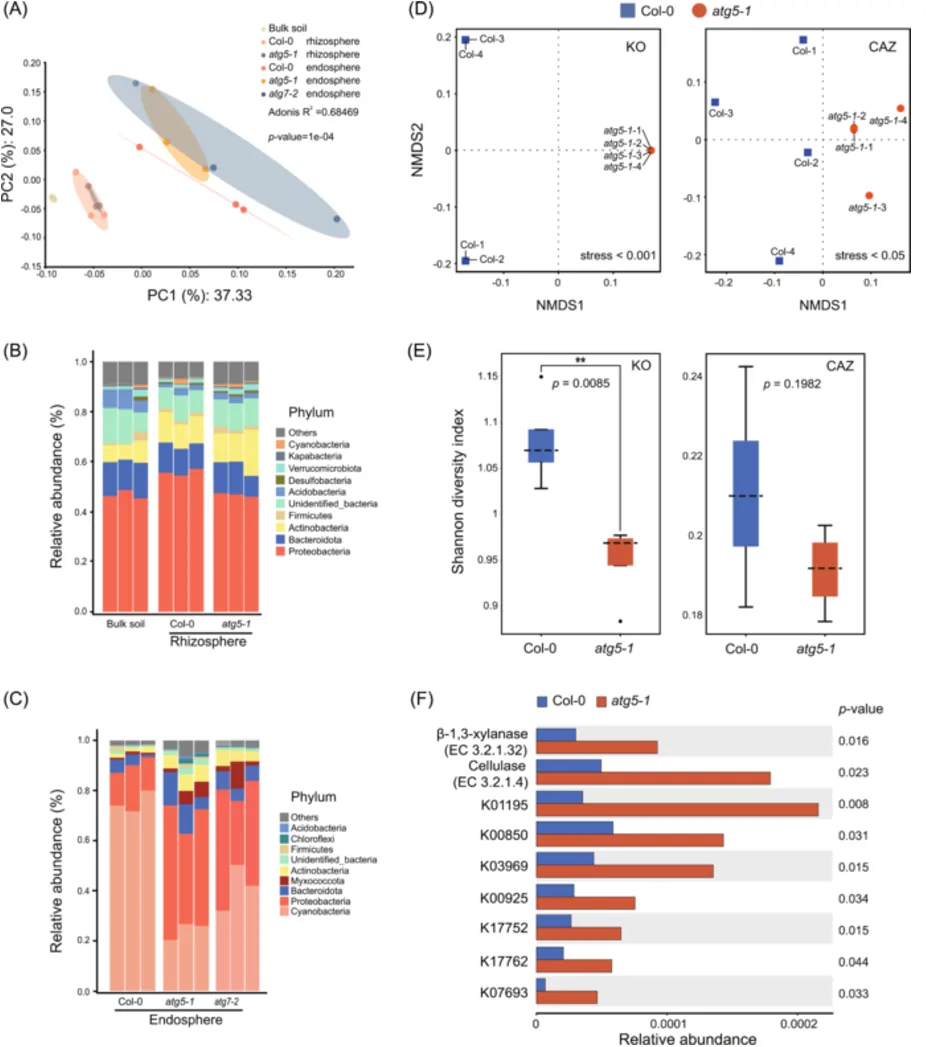
Autophagy impacts the root‐associated microbiota assembly and the functional composition of the root microbiome. (A) Principal component analysis (PCA) of bacterial communities using unweighted UniFrac metrics in the samples. The samples are depicted with different symbols, and the sites are color coded according to the compartment. (B, C) Bacterial community composition at the phylum level in the bulk soil, rhizosphere (B), and root endospheric (C) samples. (D) Nonmetric multidimensional scaling (NMDS) analysis based on KEGG ontology (KO) of KEGG and Level 2 of CAZy. *n* = 4 biological replicates. (E) Comparisons of Shannon diversity of KO and carbohydrate‐active enzymes (CAZ) functional genes between root microbiome of the Col‐0 and *atg5‐1* plants. **Indicates statistical significance (*p* < 0.01, Student's *t*‐test), *n* = 4 biological replicates. (F) Differential abundance analysis of microbiome functional genes between the Col‐0 and *atg5‐1* plants. Student's *t*‐test was performed.

To determine the factors influencing the formation of the *Arabidopsis* root microbiome, we first explored the effects of compartments on the soil microbiome. Samples were harvested from the bulk soil (unplanted pots), rhizosphere, and endospheric compartments according to a previously reported protocol [14] (Figure S1). Pooled principal component analysis (PCA) showed that the samples from different compartments formed distinct clustering patterns (Figure 1A). The decrease in the richness of the microbial population from outside to inside the root is consistent with the results from previous studies of root microorganisms, which also indicates the selectivity of plant roots to bacteria [15] (Figure S2). To gain a thorough understanding of the taxonomic structure of microbial communities in different compartments, relative abundance analyses were performed at the phylum level based on species annotation results. The phyla detected in rhizosphere samples were not consistent with those found in root samples (Figure 1B,C). Compared with the rhizosphere, the relative abundance of *Actinobacteria* was considerably lower and that of *Cyanobacteria* was substantially higher in root samples. These results illustrated the selectivity of *Arabidopsis* for microorganisms associated with roots (Figure 1B,C).

After characterizing the role of plant compartments on soil microbial assembly, we further investigated the potential impact of autophagy on plant‐microbial interactions. We observed that the difference between Col‐0 and *atg5‐1* (an autophagy mutant) in endosphere bacterial community was much greater than that in rhizosphere bacterial community (Figures 1B,C, and S3). To further confirm the effects of autophagy on endosphere bacterial community, we used another autophagy mutant, *atg7‐2*, to compare with Col‐0 in the endosphere. PCA revealed that *atg5‐1* and *atg7‐2* were clearly separated from Col‐0, indicating that autophagy mutations altered the root microbiota (Figure 1A). By comparing the taxonomic structure of the microbial community at the phylum level, *atg5‐1* and *atg7‐2* both showed increasing abundance of *Proteobacteria* and notably decreasing abundance of *Cyanobacteria* (Figure 1C). Subsequently, OTUs were clustered with 97% consistency for effective tags of all the samples to analyze the common and unique OTUs between Col‐0 and *atg5‐1* in the endosphere. As shown in the Venn diagram, *atg5‐1* contained 1531 unique OTUs, whereas Col‐0 had only 227 OTUs (Figure S3A). We detected overlap of OTUs in Col‐0 and *atg5‐1*: 847 OTUs (78.8% in Col‐0; 35.6% in *atg5‐1*). Only 773 of 847 OTUs were annotated at the phylum level (Figure S3B,C). Linear discriminant analysis Effect Size (LEfSe) analysis identified bacteria with significant differences in abundance in the root microbial community. Col‐0 increased the abundance of o_*Veillonellales_Selenomanadales*, c_*Negativicutes*, f_*Sporamusaceae*, f_*xanthobacteraceae*, o_*Rhizobiales*, and f_*Burkholderiaceae*, whereas *atg5‐1* only increased the abundance of o_*Xanthomonadales* and f_*Rhodanobacteraceae* (Figure S3D). Together, these results indicated that autophagy affects the diversity of the root microbiota.

Furthermore, we employed metagenomic analysis to explore the functional shift in the root‐associated microbiomes that might be induced by autophagy in terms of microbial genes. The analysis showed that the functional composition of the root microbiome of *atg5‐1* (i.e., Nonmetric multidimensional scaling [NMDS] ordinations of KEGG ontology [KO] and carbohydrate‐active enzymes [CAZ]) was significantly altered compared with that of Col‐0 (Figure 1D). The KEGG analysis results also showed that the absence of autophagy reduced the functional diversity of the root microbiome (Figure 1E). In addition, the total reads were statistically similar between the Col‐0 and *atg5‐1* root samples (Figure S4). The proportion of microbial DNA in *atg5‐1* was significantly higher than that in Col‐0, which is consistent with the results from the analysis of the Shannon diversity using 16S data (Figure S2). Moreover, a CAZy database analysis showed that the relative levels of cellulase (EC 3.2.1.4) and beta‐1,3‐xylanase (EC 3.2.1.32) were higher in *atg5‐1* than in Col‐0, and their higher levels may play a positive role in bacteria entering the plant roots (Figure 1F). GO annotation analysis was done on all microbial genes after removing the reads of from plant DNA. A total of 29,834 genes (50.9% of the total genes) were identified to be responsible for metabolism. Of these, most microbial genes were primarily linked to carbohydrate metabolism (Figure S5). Comparison of microbial DNA between Col‐0 and *atg5‐1* revealed alterations in the abundance of several microbial genes related to carbohydrate metabolism (Figure 1F and Table S1). Moreover, the functions of 7276 genes (12.4% of total genes) were related to environmental information processing, such as signal transduction and membrane transport, which are important biological processes for cell adaptation and survival (Figure S5). These findings indicated that the microorganisms in the *atg5‐1* roots face a more complex environment than those of Col‐0.

**Figure 2:**
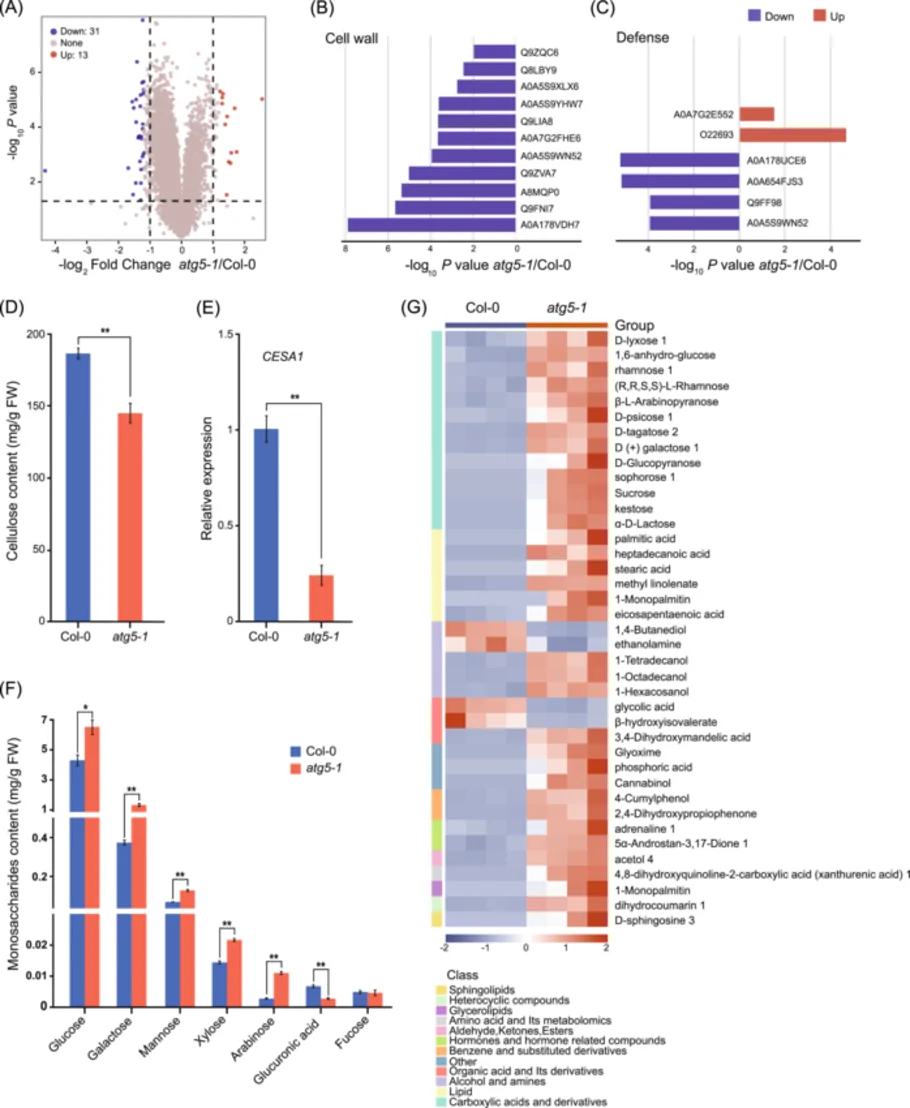
Exploring the role of autophagy in plant‐microbe interactions by proteomic and metabolomic analyses. (A) Volcano plots showing the preferential accumulation of individual proteins in roots of *atg5‐1* versus Col‐0 plants. Each protein was plotted according to its fold change in abundance (*atg5‐1*/Col‐0) and its −log_2_ *p*‐value based on three biological replicates. (B, C) Enrichment analyses of differentially expressed proteins associated with cell wall and defense. Each differentially expressed protein was plotted according to its log_10_‐fold enrichment/depletion based on three biological replicates. (D) Quantification of cellulose in 5‐week‐old Col‐0 and *atg5*‐1 roots. (E) Quantitative real‐time polymerase chain reaction (qRT‐PCR) measurements of *Arabidopsis CESA1* gene expression levels. Each bar represents the mean (±SD) gene expression levels from three biological replicates. Asterisks indicate significant differences between wild‐type Col‐0 and the *atg5‐1* mutant revealed by Student's *t*‐test. *Indicates *p* < 0.05, **indicates *p* < 0.01. (F) Monosaccharide composition in the cell walls of 5‐week‐old Col‐0 and *atg5‐1* roots. Each bar represents the mean (±SD) monosaccharide content from three biological replicates. Asterisks indicate significant differences between wild‐type Col‐0 and the *atg5‐1* mutant revealed by Student's *t*‐test. *p < 0.05, **p < 0.01. (G) Heatmap of differences metabolites. Sample names are presented horizontally and differential metabolite information is presented vertically. Different colors are filled with different values obtained after standardization of different relative contents (red represents high content, blue represents low content).

Autophagy is known to be a protein degradation pathway, and we hypothesized that the absence of autophagy can alter the content of certain proteins and thereby regulates plant‐root microbiota interactions. Therefore, we examined the potential links of plant root proteome and metabolism to autophagy‐dependent substance recycling homeostasis. The volcano plot showed 44 differential expression proteins (DEPs) between the *atg5‐1*/Col‐0 comparison groups, and the asymmetric dot distribution indicated that the absence of autophagy had a significant impact on the *Arabidopsis* root proteome (Figure 2A and Table S2) (*p* < 0.05 and FC > l1.2 l). The results from the biological function analysis using the UniProt database revealed that these DEPs are mainly related to cell wall and defense (Figure 2B,C). In 11 DEPs associated with cell walls, there are seven DEPs directly relating to the components of the cell wall (A0A7G2FHE6, A0A5S8XLX6, Q9LIA8, Q9ZQC6, A0A178VDH7, Q9FNI7, and Q8LBY9) (Figure 2B). In defense‐related DEP clusters, four proteins (A0A178UCE6, Q9FF98, A0A654FJS3, and A0A5S8WN52) were enriched in Col‐0, and two proteins (O22693 and A0A7G2E552) were enriched in *atg5‐1* (Figure 2C). In conclusion, the proteome analysis further indicated that the variation in the root microbial population in autophagy mutants was mainly caused by plant cell wall and defense‐related processes.

Following the protein hints of altered cell wall composition, we measured the contents of cellulose in the root samples. The results showed that the cellulose levels of *atg5‐1* were decreased by 22% (Figure 2D). The lower cellulose content suggested that *atg5‐1* may have disruptions in cellulose biosynthesis. Congruent with the proteomic findings, quantitative RT‐PCR demonstrated repressed gene expression of cellulose synthase 1 (CESA1), which is a major cellulose synthase in *Arabidopsis* in *atg5‐1* compared to Col‐0. (Figure 2E). These findings implied that autophagy might control the synthesis of cellulose.

Plant cell wall mechanics are primarily determined by cellulose, but hemicelluloses and pectins also have an effect on wall mechanics and cell expansion, respectively [16]. Five‐week‐old Col‐0 and *atg5‐1* roots were subjected to standard cell wall biochemical analysis to investigate potential changes in noncellulosic matrix polysaccharides (Figure 2F). We found that the contents of mannose (Man), arabinose (Ara), galactose (Gal), xylose (Xyl), and glucose (Glu) were significantly increased in *atg5‐1* roots relative to Col‐0 controls (Figure 2F). It was evident from these data that *atg5‐1* modified matrix polysaccharide abundance in addition to affecting cellulose biosynthesis.

To gain more insights into how autophagy affects the root microbiota, we analyzed differences in root exudates between wild‐type and autophagy mutant by GC‐MS. The results revealed 39 differentially accumulated metabolites between Col‐0 and *atg5‐1* (*p* < 0.05 and fold change > l2l). The comparison of *atg5‐1* with Col‐0 revealed 35 upregulated metabolites and four downregulated metabolites (Table S3). Carboxylic acids and derivatives accounted for the largest proportion of differentially accumulated metabolites (33.33%), followed by lipids (15.38%) (Figures 2G and S6). These findings suggested that autophagy induces significant changes in root exudates and the absence of autophagy leads to increased accumulation of carbohydrates and lipids. By KEGG enrichment analysis, 39 different metabolites were annotated in 27 metabolic pathways. The most noteworthy enrichment pathway was the biosynthesis of unsaturated fatty acids (ko01040), followed by fatty acid synthesis (ko00061) and fatty acid degradation (ko00071) (Figure 2G). Therefore, autophagy may exert a stronger effect on lipid metabolism.

In conclusion, we utilized a multi‐omics approach to reveal that autophagy comprehensively regulates *Arabidopsis* root microbial assembly through plant cell wall, defense, and root exudates. These results link plant protein metabolic pathways with the root microbiome and thereby not only provide insights into the mechanisms of plant and root microbial interactions but also emphasize the complexity of autophagy regulation in plants.

## AUTHOR CONTRIBUTIONS

**Shan Cheng:** Conceptualization; methodology; investigation; formal analysis; Writing—original draft. **Yunfeng Shi:** Formal analysis; writing—review and editing. **Weiming Hu:** Writing—review and editing. **Fen Liu:** Conceptualization; writing—review and editing; funding acquisition. All authors have read the final manuscript and approved it for publication.

## CONFLICT OF INTEREST STATEMENT

The authors declare no conflict of interest.

## Supporting information

Figure S1 Scheme of sample harvesting.
Figure S2 Shannon diversity of the root microbial community.
Figure S3 OTU analysis of Col‐0 and *atg5‐1*.
Figure S4 Read counts of the metagenomic sequencing.
Figure S5 KEGG pathway annotation analysis of metagenomic functional genes.
Figure S6 KEGG enrichment analysis of differential metabolites.

Table S1 Functional annotation of differentially abundant microbial genes between Col‐0 and *atg5‐1* (*p* < 0.05 and log2 [FC] > 0.6).
Table S2 Differentially expressed proteins (DEPs) between Col‐0 and *atg5‐1*. Table S3 Analysis of root exudates components between Col‐0 and *atg5‐1*.

## Data Availability

The raw sequencing data have been deposited in the National Genomics Data Center (NGDC), Beijing Institute of Genomics, and Chinese Academy of Sciences/China National Center for Bioinformation (https://ngdc.cncb.ac.cn/), under the accession number CRA012049 (16S rRNA amplicons, https://ngdc.cncb.ac.cn/gsa/s/aR0txtV8), CRA012039 (metagenomic data, https://ngdc.cncb.ac.cn/gsa/s/X1MfxAj0), and OMIX004656 (proteomic data, https://ngdc.cncb.ac.cn/omix/preview/0Npy6f3d) respectively. The data and scripts used are saved in GitHub https://github.com/576016428/Cheng-shan. Supplementary materials (methods, figures, tables, graphical abstract, slides, videos, Chinese translated version and update materials) may be found in the online DOI or iMeta Science http://www.imeta.science/imetaomics/.
