# Supplementary material for: Arabidopsis multiomics reveals the role of autophagy in root microbiome assembly
Source: IMetaOmics. 2024 Sep 5;1(2):e28. doi: 10.1002/imo2.28 (PMC12806256; doi:10.1002/imo2.28)
Supplement: Supplementary file 1 — Figure S1 Scheme of sample harvesting. Figure S2 Shannon diversity of the root microbial community. Figure S3 OTU analysis of Col‐0 and atg5‐1. Figure S4 Read counts of the metagenomic sequencing. Figure S5 KEGG pathway annotation analysis of metagenomic functional genes. Figure S6 KEGG enrichment analysis of differential metabolites. [file IMO2-1-e28-s001.docx]

**Supporting information** to: *Arabidopsis* multi-omics reveals the role of autophagy in root microbiome assembly

Running title: Autophagy affects root microbiome assembly.

Shan Cheng^#^, Yunfeng Shi^#^, Weiming Hu^*^, Fen Liu^*^

Lushan Botanical Garden, Jiangxi Province and Chinese Academy of Sciences, Jiujiang, China

Correspondence

^#^ These authors have contributed equally to this study.

^*^ Correspondence should be addressed to Fen Liu (Email: [liuf@lsbg.cn](mailto:liuf@lsbg.cn)) and Weiming Hu (Email: [huwm@lsbg.cn](mailto:huwm@lsbg.cn))

**This file includes:**

Supplemental Methods

Figures S1-S6

Tables S1-S3

Supplemental References

**LIST OF SUPPLEMENTAL INFORMATION**

**Supplemental Methods**

**Supplemental Figures**

**Figure S1:** Scheme of sample harvesting. Sterile scissors were used to cut off the aboveground part of *Arabidopsis*, and large pieces of soil were shaken off, leaving approximately 1 mm of soil around the roots. The rhizosphere soil was harvested from the roots by shaking them in sterile buffer. The roots were then sonicated in a new tube containing sterile buffer solution, and the cleaned-surface roots were rapidly frozen and lyophilized for further processing.

**Figure S2:** Shannon diversity of the root microbial community. Letters denote statistical significance (Student’s *t* test).

**Figure S3:** OTU analysis of Col-0 and *atg5-1*. (A) Venn diagram showing the overlap of OTUs between the Col-0 and *atg5-1* root samples. (B, C) The taxonomic composition of the overlapping OTUs between Col-0 (B) and *atg5-1* (C) at the phylum level is shown. (D) LEfSe analysis identified the microbes whose abundances significantly differed between the Col-0 and *atg5-1*. The findings with regards to kingdom (k), phylum (p), class (c), order (o), and family (f) are shown in the plot. Species with LDA greater than the set value of four are presented. The length of the bar indicates the magnitude of LDA influence (LDA score > 4, *p* < 0.05).

**Figure S4:** Read counts of the metagenomic sequencing. Stacked bars show the number of reads in plant DNA and microbial DNA sequences in root samples. Each bar represents the mean number (±SD) of sequencing reads from four biological replicates. Asterisks indicate significant differences between the wild-type Col-0 and the *atg5-1* mutant revealed by Student’s *t* test. ** indicates *p* value < 0.01; NS, not significant.

**Figure S5:** KEGG pathway annotation analysis of metagenomic functional genes. Microbial genes were annotated from six major metabolic pathways in the KEGG database. The abscissa represents the number of proteins, and the ordinate represents the annotated KEGG entries.

**Figure S6:** KEGG enrichment analysis of differential metabolites The color of the dot reflects the *p* value. The size of the dot represents the number of enriched differentially accumulated metabolites.

**Supplemental Tables (TableS.xlsx)**

**Table S1:** Functional annotation of differentially abundant microbial genes between Col-0 and *atg5-1* (*p* < 0.05 and log2 (FC) > 0.6).

**Table S2:** Differentially expressed proteins (DEPs) between Col-0 and *atg5-1*.

**Table S3:** Analysis of root exudates components between Col-0 and *atg5-1*.

**Supplemental Methods**

**Plant growth and soils**

Seeds of wild-type (Col-0), autophagy mutant *atg5-1* (SAIL_129_B07) and *atg7-2* (GABI_655B06) *Arabidopsis* were surface-sterilized and stored at 4 °C for 2 days to eliminate any dormancy and ensure uniform germination. The sterilized seeds were then germinated on 1/2 MS plates. Five-day-old seedlings were transplanted to pots filled with mixed soil, with nine seedlings per pot. All pots were placed in a growth chamber with a long-day photoperiod of 16 h of light at 21 °C and 8 h of darkness at 18 °C. The relative humidity was set to 50%. As a control, unplanted pots were placed under the same conditions.

Natural soil was harvested from the Nanchang Botanical Garden, Nanchang, China. The soil was cleaned and homogenized using a 2.5-mm sieve and then mixed with commercial soil (Pindstrup Substrate) at a 2:1 ratio.

**Experimental setup and sampling**

Samples from four pots were collected as one biological replicate. Five-week-old plants with consistent growth status were harvested for root microbiota analysis, metagenomic sequencing, proteomic sequencing, determination of cellulose content and quantitative real-time PCR (qRT-PCR).

For 16S rRNA sequencing and metagenomic sequencing, samples were prepared as described by Bulgarelli et al [1]. Bulk soil samples were collected from unplanted pots after removing the top 1 cm of soil. The aboveground plant organs were removed from each pot, and large soil aggregates were manually shaken down from the roots. Following the conventional definition of rhizosphere soil [2], approximately 1 mm of soil was still closely attached to the roots. The roots were placed in a sterile 50-mL Falcon tube with 20 mL of phosphate buffer (45.8 mM NaH_2_PO_4_·H_2_O, 61.5 mM Na_2_HPO_4_·7H_2_O, and 0.002% Silwet L-77). The tubes were washed on a shaking platform for 10 min at 200 rpm, and the roots were then transferred to a new 50-mL falcon with 20 mL of sterile phosphate buffer for 10 min at 200 rpm. The soil suspensions from the two washing treatments were combined in a Falcon tube and centrifuged at 4000 g for 20 min. The precipitate was defined as the rhizosphere compartment, frozen in liquid nitrogen and stored at -80 °C. The washed roots were transferred to another Falcon tube and sonicated at 160 W for 10 min (ten 30-s bursts followed by ten 30-s rests). After sonication, the excess water on the roots was absorbed by filter paper, transferred to 2-mL tubes and frozen in liquid nitrogen for storage at -80 °C. The sonicated root was defined as the endosphere compartment.

For proteomic sequencing and measurement of the cellulose and monosaccharide contents, the roots were washed with phosphate buffer, frozen in liquid nitrogen and stored at -80 °C until further processing.

**16S rRNA amplicon sequencing and data analysis**

The genomic DNA of the samples was extracted by cetyltrimethylammonium bromide (CTAB)/sodium dodecyl sulfate (SDS) methods and then diluted to 1 ng/μL with sterile water. The primers 799F (5’-AACMGGATTAGATACCCKG-3’) and 1193R (5’-ACGTCATCCCCACCTTCC-3’) were used to amplify the V5-V7 fragment of the 16S rRNA gene. The amplicons were sequenced on the IonS4S3™ XL platform by Novogene Co., Ltd. (Tianjin, China).

After processing the raw sequencing data, the Uparse algorithm (Uparse v7.0.1001) [3] was utilized for 16S rRNA analysis, and the sequences with ≥ 97% identity were clustered into operational taxonomic units (OTUs). The alpha- (within samples) and beta- (among samples) diversity were calculated using Quantitative Insights Into Microbial Ecology (QIIME) software [4].

**Metagenome sequencing and data analysis**

The sonicated root samples were used for metagenome sequencing. The genomic DNA (1 μg) was randomly cleaved into short fragments, end-repaired, A-tailed and further ligated with an Illumina adapter. The fragments with adapters were amplified by PCR, size selected, and purified. The library was sequenced using a NovaSeq 6000 instrument with PE150 by Novogene (Beijing, China).

The sequencing results were preprocessed and assembled using MEGAHIT software (v1.0.4-beta) [5]. MetaGeneMark (V3.05, http://topaz.gatech.edu/GeneMark/) was used to perform gene prediction. We then used Bowtie2 (Bowtie2.2.4) to calculate the abundance of each gene in each sample. According to the sequences of bacteria, fungi and viruses extracted from the NCBI NR database, the species annotation information of each sequence was determined using DIAMOND software [6]. The gene sequences were compared with the Kyoto Encyclopedia of Genes and Genomes (KEGG) database, and the relative abundance at different functional levels was calculated.

**Protein extraction, quantification, and digestion**

Ground root tissues were lysed with SDT lysis buffer (2% SDS, 100 mM DTT, 100 mM Tris-HCL, pH 7.6, and 100 mM NaCl) and 1/100 volume of DTT and then ultrasonicated for 5 min on ice. The reaction was carried out at 95 °C for 8-15 min, placed in an ice bath for 2 min and centrifuged at 12000 g for 15 min at 4 °C. The supernatant was allowed to react with sufficient iodoacetamide (IAM) for 1 h in the dark. Four times the volume of precooled acetone was added, and the sample was precipitated at -20 °C for at least 2 h. Samples were collected by centrifugation at 12000 g at 4 °C for 15 min. After rinsing with 1 mL of cold acetone, the precipitate was thoroughly dissolved in dissolution buffer [0.1 M triethylammonium bicarbonate (TEAB), pH 8.5 and 6 M urea]. A Bradford protein quantitative kit was used to measure the protein concentration. Each protein sample, containing precisely 100 μg of protein, was digested with Trypsin Gold (Promega) at 37 °C for 16 h. The peptides were then desalted using the C18 cartridge and dried by vacuum centrifugation.

**Tandem mass tag (TMT)-based quantitative proteomic profiling**

The treated peptides were labeled with TMT6/10-plex reagents (Thermo Fisher Scientific) in accordance with the manufacturer’s instructions. Then, 0.1 mL of 0.1 M TEAB buffer was used to dissolve the peptides, and 41 μL of acetonitrile-dissolved TMT labeling reagent was added. After shaking for 2 h, the reaction was stopped by the addition of 8% ammonia. All labeled samples were mixed at equal volumes, desalted and lyophilized. The mixed tagged peptides were fractionated with a Waters BEH C18 column (4.6×250 mm, 5 μm) on a Rigol L-3000 HPLC system. The mobile phases are typically referred to as “mobile phase A” (2% acetonitrile, adjusted to pH 10.0 using ammonium hydroxide) and “mobile phase B” (98% acetonitrile, adjusted to pH 10.0 using ammonium hydroxide). The gradient elution was set to the following: 0-10 min, 3-5% B; 10-30 min, 5-20% B; 30-48 min, 20-40% B; 48-50 min, 40-50% B; 50-53 min, 50-70% B; and 53-54 min, 70-100% B. The eluates were collected in a tube every minute and merged into 10 fractions based on the UV absorbance at 214 nm. The collected fractions were lyophilized and dissolved in 0.1% formic acid (FA).

Proteomics analyses were performed using an EASY-nLCTM 1200 UHPLC system coupled with a Q Exactive HF-X mass spectrometer (Thermo Fisher Scientific). A ReproSil-Pur 120 C18-AQ analytical column (15 cm×150 μm, 1.9 μm) was used to separate peptides, and a 90-min linear gradient for TMT6-plex with eluate A (0.1% FA in Ultrapure water) and eluent B (0.1% FA in 80% acetonitrile) was used. The mass spectrometer was operated in the data-dependent acquisition mode with full scans (350-1500 m/z). The resolutions of the primary and secondary mass spectrometers were 60000 (200 m/z) and 45000 (200 m/z), respectively. LC‒MS/MS testing was conducted by Novogene Co., Ltd. (Beijing, China). The resulting TMT spectra were searched against the Arabidopsis_thaliana_UniProt_2022_1_27.fasta.fasta (136782 sequences) protein database using the search engine Proteome Discoverer v2.5 r (PD, Thermo, HFX and 480). The search results were further filtered through Proteome Discoverer v2.5, and peptide spectrum matches (PSMs) with a credibility greater than 99% were identified. The identified proteins contained at least 1 unique peptide. The identified PSMs and proteins were retained and analyzed based on a false discovery rate (FDR) < 1.0%. The protein quantitation results were statistically analyzed by Student’s t test, and the DEPs were defined as those that met the following criteria: *p* < 0.05 and fold change (FC) > 1.2 or < 0.83.

Gene Ontology (GO) and InterPro (IPR) functional analyses were conducted using the InterProScan program [7] against the nonredundant protein database (including PANTHER, Pfam, ProDom, ProSite, PRINTS, and SMART), and the protein families and pathways were analyzed using the Clusters of Orthologous Groups (COG) and Kyoto KEGG databases.

**Measurement of the cellulose content**

The cellulose content in the roots of Col-0 and the *atg5-1* mutant line was determined using a plant cellulose ELISA kit (ML Bio, Shanghai, China). All experiments were repeated three times.

**Quantitative real-time PCR**

The RNA was extracted from ground root tissues of Col-0 and the *atg5-1* mutant line using a FastPure Universal Plant Total RNA Isolation Kit (Vazyme), and was subjected to DNA removal and reverse transcription using a FastQuant RT kit with gDNase (TIANGEN). qRT–PCR was carried out using iTaq Universal SYBR Green PCR master mix (Bio-Rad) and a CFX96 real-time PCR detection system (Bio-Rad). The primers used in this study are actin2-F (5’-GGTAACATTGTGCTCAGTGGTGG-3’), actin2-R (5’-AACGACCTTAATCTTCATGCTGC-3’), CESA1-F (5’-GAGCTGAGATGGAGCGGTGT-3’) and CESA1-R (5’-CTGCTCGTTCCTCCACCAAT-3’).

**Analysis of root exudates**

Seeds of Col-0 and *atg5-1* were surface-sterilized and stratified at 4 °C for 48 h. Seedlings were grown for 7 days in liquid MS medium under a 16-h light/8-h dark photoperiod at 21 °C and then transferred to fresh MS medium lacking sucrose. After an additional 10 days, the medium was collected and filtered through a 0.45-μm PVDF membrane. The filtered exudates were flash-frozen in liquid nitrogen, lyophilized and maintained at -80 °C. MS medium without sucrose was used as a control.

The relative quantities of root metabolites in the Col-0 and *atg5-1* samples were analyzed with an ultrahigh-performance liquid chromatography-electrospray ionization-tandem mass spectrometry system (UPLC-MS), and the polysaccharide contents were detected using an Agilent 8890 gas chromatograph coupled to a 5977B mass spectrometer (GC-MS) platform by MetWare (Wuhan, China). The lyophilized sample was vortexed to aspirate 30 mL of concentrated dry product, and 3 mL of the extract was added. After mixing, 1 mL of sample was moved to a 2-mL centrifuge tube, and 0.02 mL of internal standard (10 μg/mL) was added. The mixture was blown dry with a nitrogen blower and lyophilized in a freeze-dryer, and 0.1 mL of pyridinium methoxide salt (0.015 g/mL) and oxime was then added. The resulting mixture was incubated in an oven at 37 °C for 2 h. Then, 0.1 mL of bis(trimethylsilyl)trifluoroacetamide (BSTFA) (with 1% chlorotrimethylsilane) was added, and the mixture was reacted in an oven at 37 °C for 30 min to obtain the derivatization solution. The solution was diluted to 1 mL by adding hexane, filtered through a 0.22-μm organic-phase needle filter, and stored at -20 °C. An Agilent gas chromatograph coupled to a mass spectrometer with a DB-5MS column (30 m length×0.25 mm i.d.×0.25 μm film thickness, J&W Scientific, USA) was employed for GC-MS analysis of the extracting solution. Helium was used as the carrier gas at a flow rate of 1.2 mL/min. Injections were made in the front inlet mode with a split ratio of 5:1, and the injection volume was 1 μL. The oven temperature was held at 40 °C for 1 min, increased to 100 °C at 20 °C/min, increased to 300 °C at 15 °C/min, and held at 300 °C for 5 min. All samples were analyzed in the scan mode. The ion source and transfer line temperatures were 230 °C and 280 °C, respectively.

**Analysis of the monosaccharide composition**

The freeze-dried samples were crushed with a zirconia bead at 30 Hz for 1.5 min using a mixer mill (MM 400, Retsch). Twenty milligrams of powder was collected, diluted to a final volume of 500 μL with methanol:isopropanol:water (3:3:2, V/V/V), vortexed for 3 min and ultrasonicated for 30 min. After centrifugation at 12000 rpm for 3 min at 4 °C, 12.5 μL of the supernatant was mixed with 20 μL of the internal standard (250 μg/mL) and evaporated under a nitrogen gas stream. The evaporated sample was transferred to a lyophilizer for freeze-drying. The residue was used for further derivatization. The derivatization method was as follows: the sample was mixed with 100 μL of a solution of methoxyamine hydrochloride in pyridine (15 mg/mL). After incubation at 37 °C for 2 h, 100 μL of BSTFA was added, and the mixture was vortexed, maintained at 37 °C for 30 min, diluted to an appropriate concentration and analyzed by GC-MS. An Agilent 7890B gas chromatograph coupled to a 7000D mass spectrometer with a DB-5MS column was used for the analysis of sugars. Helium was used as the carrier gas at a flow rate of 1 mL/min. Injections were made in the split mode with a split ratio of 3:1, and the injection volume was 2 μL. The oven temperature was maintained at 150 °C for 1 min, increased to 200 °C at 5 °C/min, 300 °C at 16 °C/min, and 320 °C at 20 °C/min and held at the final temperature for 5.5 min. All samples were analyzed in the selective ion monitoring mode. The ion source and transfer line temperatures were 230 °C and 280 °C, respectively.

**
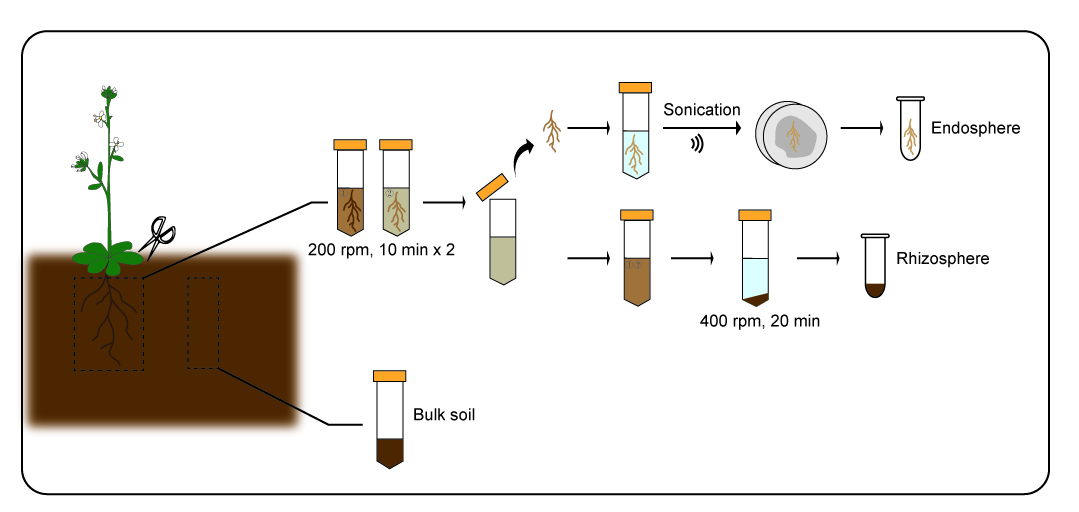
**

**Figure S1.** Scheme of sample harvesting. Sterile scissors were used to cut off the aboveground part of *Arabidopsis*, and large pieces of soil were shaken off, leaving approximately 1 mm of soil around the roots. The rhizosphere soil was harvested from the roots by shaking them in sterile buffer. The roots were then sonicated in a new tube containing sterile buffer solution, and the cleaned-surface roots were rapidly frozen and lyophilized for further processing.

**
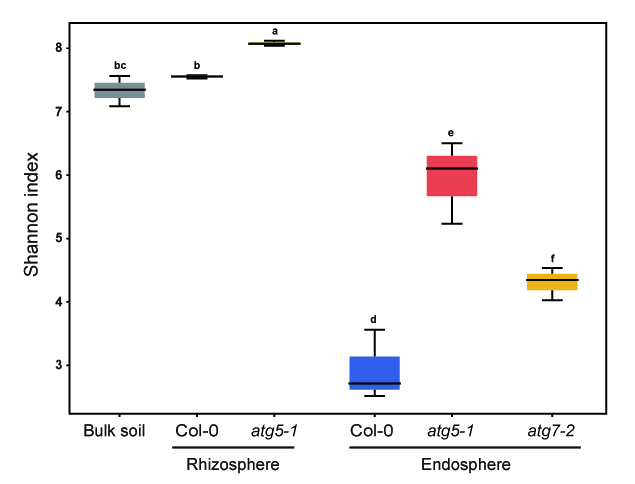
**

**Figure S2.** Shannon diversity of the root microbial community.Letters denote statistical significance (Student’s *t* test).


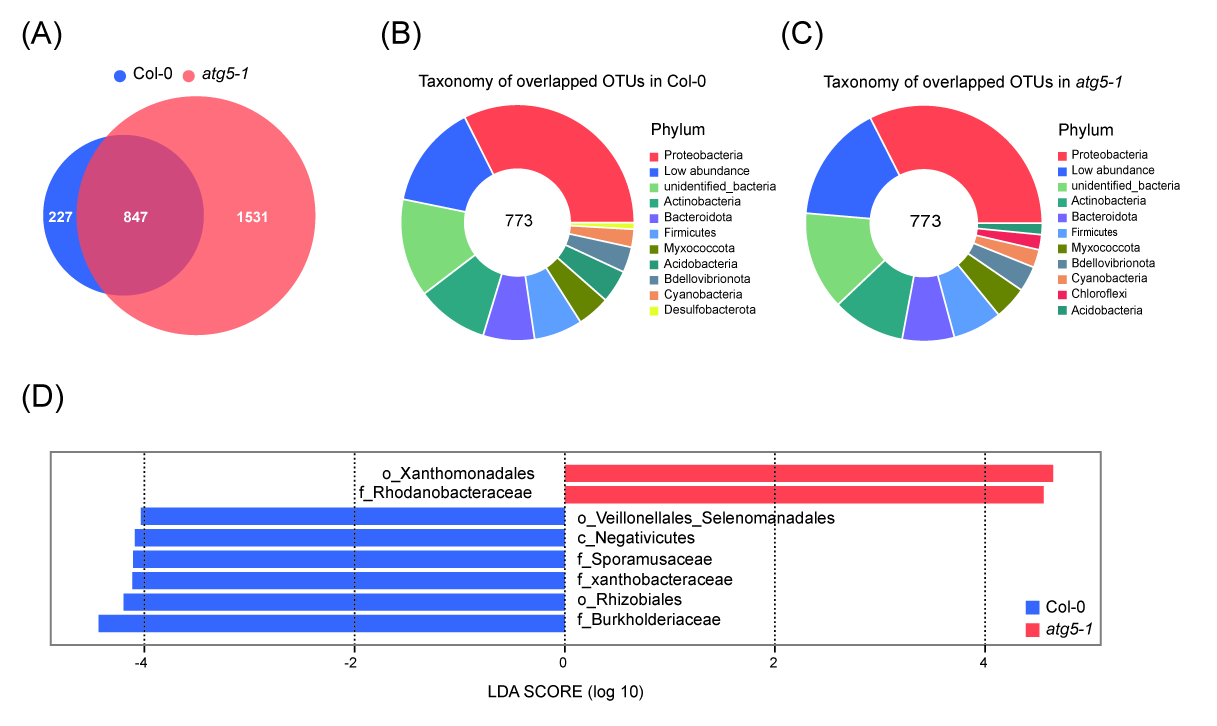


**Figure S3.** OTU analysis of Col-0 and *atg5-1*. (A) Venn diagram showing the overlap of OTUs between the Col-0 and *atg5-1* root samples. (B, C) The taxonomic composition of the overlapping OTUs between Col-0 (B) and *atg5-1* (C) at the phylum level is shown. (D) LEfSe analysis identified the microbes whose abundances significantly differed between the Col-0 and *atg5-1*. The findings with regards to kingdom(k), phylum(p), class (c), order (o), and family (f) are shown in the plot. Species with LDA greater than the set value of four are presented. The length of the bar indicates the magnitude of LDA influence (LDA score > 4, *p* < 0.05).

**
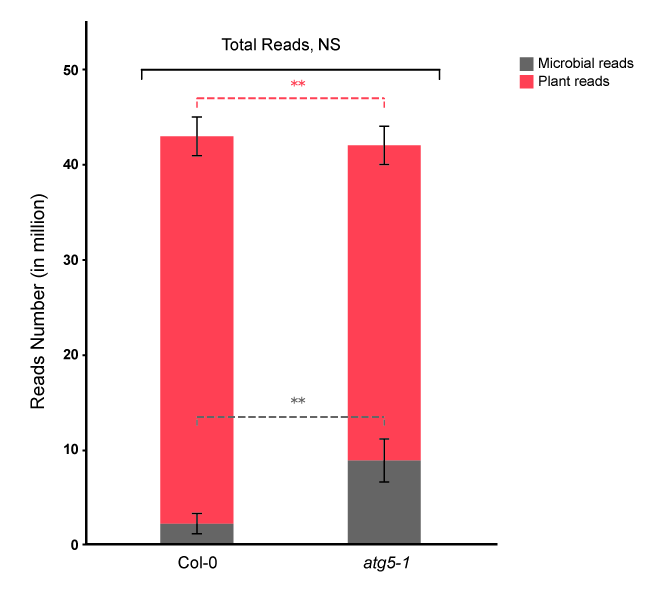
**

**Figure S4.** Read counts of the metagenomic sequencing. Stacked bars show the number of reads in plant DNA and microbial DNA sequences in root samples. Each bar represents the mean number (±SD) of sequencing reads from four biological replicates. Asterisks indicate significant differences between the wild-type Col-0 and the *atg5-1* mutant revealed by Student’s *t* test. ** indicates *p* value < 0.01; NS, not significant.


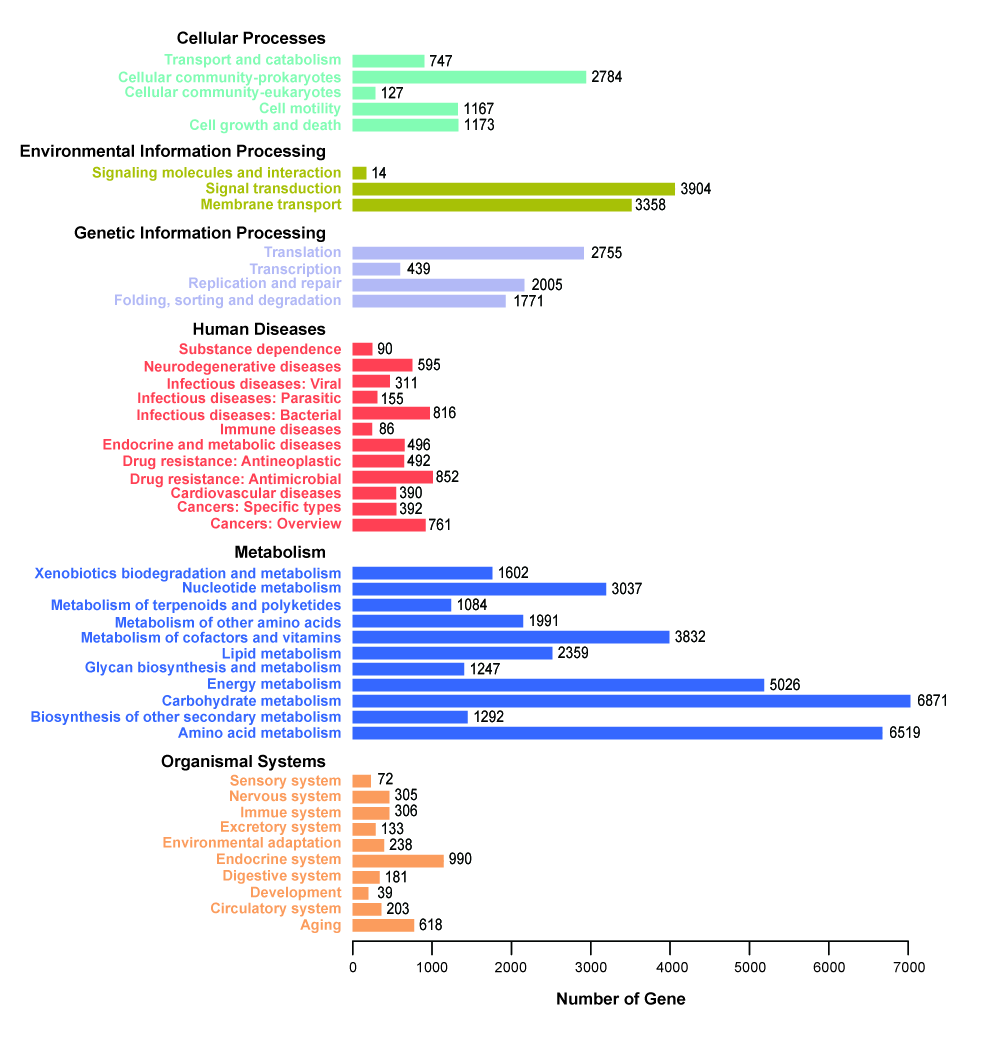


**Figure S5.** KEGG pathway annotation analysis of metagenomic functional genes. Microbial genes were annotated from six major metabolic pathways in the KEGG database. The abscissa represents the number of proteins, and the ordinate represents the annotated KEGG entries.

**
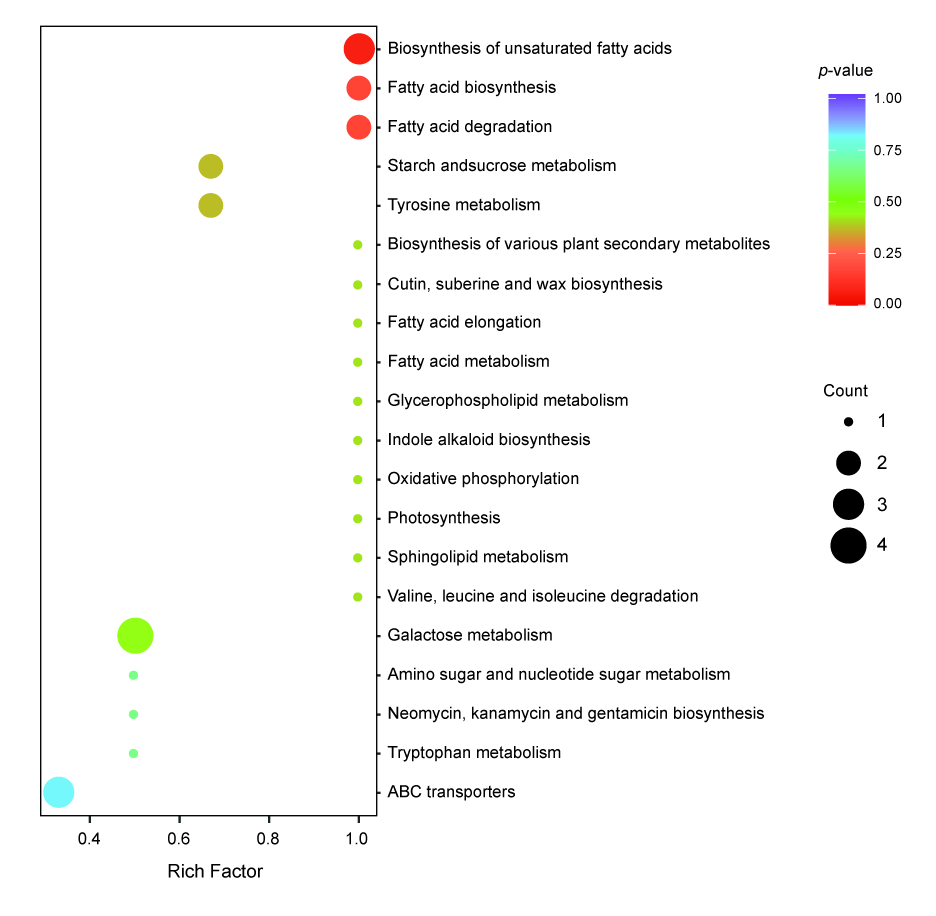
**

**Figure S6.** KEGG enrichment analysis of differential metabolites. The color of the dot reflects the *p* value. The size of the dot represents the number of enriched differentially accumulated metabolites.

**Supplemental References**

1. Bulgarelli D, Rott M, Schlaeppi K, Ver Loren van Themaat E, Ahmadinejad N, Assenza F, Rauf P, et al. 2012. “Revealing structure and assembly cues for Arabidopsis root-inhabiting bacterial microbiota.” *Nature* 488: 91-95. http://doi.org/10.1038/nature11336.

2. Troxler, Azelvandre, Zala, Defago, Haas. 1997. “Conjugative Transfer of Chromosomal Genes between Fluorescent Pseudomonads in the Rhizosphere of Wheat.” *Applied & Environmental Microbiology*, 1:213-219. http://doi.org/10.1128/aem.63.1.213-219.1997.

3. Edgar, C. Robert. 2013. “UPARSE: highly accurate OTU sequences from microbial amplicon reads.” *Nature Methods* 10:996-998. http://doi.org/10.1038/nmeth.2604.

4. Kuczynski, J., J. Stombaugh, W. A. Walters, A González, J. G. Caporaso, R. Knight. 2011. “Using QIIME to Analyze 16S rRNA Gene Sequences from Microbial Communities.” *Current Protocols in Bioinformatics* 36. http://doi.org/10.1002/9780471729259.mc01e05s27.

5. Feng, Qiang, Suisha Liang, Huijue Jia, Andreas Stadlmayr, Longqing Tang, Zhou Lan, Dongya Zhang, Huihua Xia, Xiaoying Xu, Zhuye Jie. 2015. “Gut microbiome development along the colorectal adenoma-carcinoma sequence.” *Nature Communications* 6: 6528. http://doi.org/10.1038/ncomms7528.

6. Huson, Daniel H, Suparna Mitra, Hans Joachim Ruscheweyh, Nico Weber, Stephan C Schuster. 2011. “Integrative Analysis of Environmental Sequences Using MEGAN4.” *Genome Research* 21: 1552-1560. http://doi.org/10.1101/gr.120618.111.

7. Philip, Jones, Binns David, Chang Hsin-Yu, Fraser Matthew, Li Weizhong, Mc Anulla Craig, Mc William Hamish, Maslen John, Mitchell Alex, Nuka Gift. 2014. “InterProScan 5: genome-scale protein function classification.” *Bioinformatics (Oxford, England)* 1236-1240. http://doi.org/10.1093/bioinformatics/btu031.
